# How heterogeneous are MSM from Brazilian cities? An analysis of sexual behavior and perceived risk and a description of trends in awareness and willingness to use pre-exposure prophylaxis

**DOI:** 10.1186/s12879-019-4704-x

**Published:** 2019-12-19

**Authors:** Thiago S. Torres, Luana M. S. Marins, Valdilea G. Veloso, Beatriz Grinsztejn, Paula M. Luz

**Affiliations:** Fundação Oswaldo Cruz, Instituto Nacional de Infectologia Evandro Chagas, Av. Brasil 4365, Manguinhos, Rio de Janeiro, 21040-900 Brazil

**Keywords:** MSM, HIV risk behavior, HIV perceived risk, Web-based survey, PrEP, Brazil, Latin America

## Abstract

**Background:**

Brazil has the largest population of individuals living with HIV/AIDS in Latin America, with a disproportional prevalence of infection among gays, bisexuals and other men who have sex with men (MSM). Of relevance to prevention and treatment efforts, Brazilian MSM from different regions may differ in behaviors and risk perception related to HIV.

**Methods:**

We report on MSM living in 29 different cities: 26 Brazilian state capitals, the Federal District and two large cities in São Paulo state assessed in three web-based surveys (2016–2018) advertised on Grindr, Hornet and Facebook. Using logistic regression models, we assessed the association of risk behavior with HIV perceived risk as well as factors associated with high-risk behavior.

**Results:**

A total of 16,667 MSM completed the survey. Overall, MSM from the North and Northeast were younger, more black/mixed-black, of lower income and lower education compared to MSM from the South, Southeast and Central-west. Though 17% had never tested for HIV (with higher percentages in the North and Northeast), condomless receptive anal sex (previous 6 months) and high-risk behavior as per HIV Incidence Risk scale for MSM were observed for 41 and 64%, respectively. Sexual behavior and HIV perceived risk had low variability by city and high-risk behavior was strongly associated with high HIV perceived risk. Younger age, being gay/homosexual, having a steady partner, binge drinking, report of sexually transmitted infection (STI) and ever testing for HIV were associated with increased odds of high-risk behavior. Awareness and willingness to use PrEP increased from 2016 to 2018 in most cities.

**Conclusions:**

Overall, MSM socio-demographic characteristics were heterogeneous among Brazilian cities, but similarities were noted among the cities from the same administrative region with a marked exception of the Federal District not following the patterns for the Central-West. Combination HIV prevention is most needed among young men who self-identify as gay/homosexual, report binge drinking or prior STI.

## Background

Brazil is the largest country in Latin America and the fifth largest country by area and population, with more than 212 million inhabitants [1]. Brazil is divided into five geographic regions: North (7 states), Northeast (9 states), Central-west (3 states and the Federal District), Southeast (4 states) and South (3 states). The Southeast, where São Paulo and Rio de Janeiro are located, is the most populous and industrialized region accounting for 42% of all Brazilians and almost 50% of the country’s gross domestic product (GDP). Brazilian geographic regions have impressive disparities in terms of GDP per capita: the Northeast and North have the lowest values (USD 4000-5000) while GDP per capita in the Central-west, South and Southeast ranges from USD 9000 to 10,000. Disparities in the human development index (HDI) are also observed: while HDI ranges from 0.850 to 0.766 in the states of Central-west, South and Southeast, it varies from 0.752 to 0.683 in the North and Northeast. Furthermore, disparities exist within regions and cities, as a result of the dramatic inequalities in wealth and health. In Rio de Janeiro city, for example, the human development index (HDI) varies from very high to low in two neighbor areas: Gávea (0.970) and Rocinha (0.732) [2].

Brazil has the largest population of individuals living with HIV/AIDS in Latin America, with a disproportional prevalence of infection among men who have sex with men (MSM) [3, 4]. A respondent driven sampling (RDS) study of HIV prevalence found that 18.4% of MSM in Brazil were living with HIV in 2016 [5], higher than the previous RDS study conducted in 2009 (14.2%) [6]. In 2017, approximately 50% of reported HIV infections among male were attributed to male-to-male sexual contact [7], even though a national survey has showed that approximately 3.5% (95% confidence interval [CI] 2.9–4.3%) of the Brazilian men between 15 and 64 years old reported sex with other men [8]. Unfortunately, new infections in this population continue to rise [7]. To stop the HIV epidemic in Brazil, a continental, diverse and unequal country, it is necessary to understand and verify how heterogeneous are MSM from each region are in terms of sexual behavior and risk perception.

Since December 2017, pre-exposure prophylaxis (PrEP) with emtricitabine and tenofovir disoproxil fumarate (FTC/TDF) is offered free of charge through the Brazilian Public Health System (SUS) to populations at substantial risk for HIV infection including eligible MSM within the HIV prevention package [9]. During 2018, the first year of PrEP provision in SUS, PrEP uptake among eligible MSM varied considerably from a maximum of 25% in Florianopolis (in the South) to a low of 1% in Belém (in the North) [4]. One explanation for such a discrepancy could be a lack of perceived risk that differentially impacts PrEP uptake throughout the country. Data on sexual behavior, risk perception and willingness to use PrEP among MSM from each Brazilian region is essential to support the implementation and roll-out of the PrEP program.

This study provides an analysis of sexual behavior and perceived risk, as well as a description of socio-demographic characteristics and trends in awareness and willingness to use PrEP among MSM living in all Brazilian state capitals, the Federal District and two large cities from São Paulo state (Campinas and Santos).

## Methods

### Study design

We conducted three cross-sectional web-based studies targeting MSM in Brazil from 2016 to 2018, one per year. Individuals who met eligibility criteria (age ≥ 18 years, cisgender men, and HIV uninfected self-report) and who acknowledged reading the informed consent text were directed to the online questionnaire, which was programmed on SurveyGizmo®. The first study (2016 survey) was conducted in July 2016 in 10 Brazilian capitals, two from each Brazilian Geographical Region: Belém and Manaus (North); Salvador and Recife (Northeast); Brasília and Goiânia (Central-West); Florianópolis and Porto Alegre (South); and Rio de Janeiro and São Paulo (Southeast) [10]. The second (2017 survey) [11] and third (2018 survey) [12] studies were conducted in July 2017 and March to April 2018, respectively, and were expanded to all Brazilian state capitals and two large urban centers in São Paulo State (Santos and Campinas). The 2016 and 2018 surveys were advertised on two geosocial networking (GSN) apps for sexual encounters among MSM: Hornet and Grindr. The 2018 survey was also advertised on Facebook social media. The 2017 survey was advertised on Hornet only. No incentives were provided for answering the survey and, on average, participants took approximately 10 mins to complete it.

### Survey instrument

The survey instrument was composed of five sections (25 questions) addressing: sociodemographic information, substance use, sexual behavior and history of sexually transmitted infections, HIV perceived risk and use of HIV testing as well as awareness and willingness to use PrEP (Additional file 1). Though the instrument was not the same in the three surveys, the items used in this analysis were the present in all.

### Variables

#### Socio-demographic

Age at the time of the survey was categorized in 4 brackets: 18 to 24; 25 to 29; 30 to 34 and ≥ 35 years; race was categorized in white/Asian, black and pardo (mixed-race) /native; schooling was categorized in < 10 years, 10–12 years, 13–16 years and > 16 years. Family monthly income was grouped into the following strata: ≤ 1 > 1–3, > 3–10 and > 10 minimum wages (Brazilian minimum wage was R$998 or US$268 in January 2019). Sexual orientation was dichotomized in gay or homosexual and other (bisexual, heterosexual or other). Steady partner (male or female) was dichotomized in yes/no. To infer the size of the MSM community in each city, we used the most recent nation-wide survey of sexual practices and behavior that estimated that 3.5% of men between 15 and 64 years had sex with other men [8].

#### Substance use

Binge drinking [13] was evaluated with the question “In the last 6 months, did you drink 5 or more drinks in a couple of hours?”. Use of stimulants (cocaine, poppers, crack, or amphetamines) during the previous 6 months was dichotomized in yes/no.

#### Sexual behavior and sexually transmitted infections

Sexual behavior in the last 6 months was assessed with the following questions: number of partners, condomless receptive anal sex, sex with HIV+ partner and number of insertive anal intercourses with HIV+ partner. These questions (in addition to other questions regarding age and use of stimulants) compose the HIV Incidence Risk Index for MSM (HIRI-MSM), a 7-item questionnaire developed by Smith et al. [14] to predict HIV seroconversion among MSM that is recommended by the Centers for Disease Control and Prevention (CDC) to screen individuals who should be evaluated for PrEP use [15]. Scores < 10 and ≥ 10 were considered as “low risk” and “high risk”, respectively [14, 15]. Report of sexually transmitted infections (STI; syphilis, gonorrhea or rectal chlamydia) in the last 6 months were dichotomized in yes/no.

#### HIV perceived risk and HIV testing

HIV perceived risk was assessed with the question “In your opinion, what is your risk of getting HIV in the next year?” with five possible options: “No risk”, “Low risk”, “High risk/50%”, “Certain/100%” and “I don’t know or I don’t want to answer”, which was considered as a missing value for the analysis. We have described HIV perceived risk results in three groups: “No risk”, “Low risk” and “High risk” which included the categories “High risk/50%” and “Certain/100%”). For the logistic models, “No risk” and “Low risk” were grouped into one category. Individuals were asked about previous HIV tests (never or at least once in lifetime). Additionally, among those who reported never having tested, we accessed their reasons with the question “Why have you never tested for HIV?” with the following possible responses: “I am not at risk of getting infected”, “I don’t think it is practical to go to a health care center”, “I am ashamed”, “I am afraid of getting a positive result”, “I am too lazy” and “Other reasons”.

#### Awareness and willingness to use PrEP

PrEP awareness was assessed with the question “Have you ever heard of PrEP?” (yes/no). Willingness to use PrEP was defined as the “High interest” option on a four-point Likert scale with the question “What level of interest would you have in using PrEP?”. A brief explanation about PrEP was provided before these questions were asked. These questions have been previously used by our research team to describe PrEP awareness and willingness to use PrEP [10–12, 16].

#### Statistical analysis

Socio-demographic characteristics and substance use, sexual and preventive behaviors of the participants were described according to their hometown for each Brazilian State capital, the Federal District, and two major cities in São Paulo. The results were organized according to 1) Brazilian geographical regions, from North to South, and, within each region, 2) by cities with the greatest number of participants: North (Manaus, Belém, Porto Velho, Palmas, Boa Vista, Rio Branco and Macapá), Northeast (Recife, Salvador, Fortaleza, Natal, João Pessoa, Aracaju, Teresina, Maceió and São Luís), Central-west (Brasília, Goiânia, Cuiabá and Campo Grande), Southeast (São Paulo, Rio de Janeiro, Belo Horizonte, Campinas, Vitória and Santos) and South (Porto Alegre, Curitiba and Florianópolis). Reasons for never testing for HIV were presented according to the Brazilian geographical region. Using a logistic regression model, we tested the association between an individual’s HIV perceived risk and their reported behavior as measured by: 1) the HIRI-MSM, 2) condomless receptive anal sex and 3) number of male partners. Following, we tested, for 11 cities, the hypothesis that sexual behavior as defined by the HIRI-MSM and by condomless receptive anal sex is associated with HIV risk perception using Pearson’s chi-squared test. Finally, a logistic regression model was used to explore factors associated with high risk behavior as per HIRI-MSM. Models were developed for the whole country and for the two cities with the greatest number of participants within each region (except for the Southeast, the most populous region in Brazil, with 3 cities): North (Manaus and Belém), Northeast (Recife and Salvador), Central-west (Brasília and Goiânia), Southeast (São Paulo, Rio de Janeiro and Belo Horizonte that ranks third in number of participants in the present study) and South (Porto Alegre and Curitiba). Finally, trends in PrEP awareness and willingness from 2016 to 2018 were graphically provided for the overall sample (Brazil) and the 10 state capitals included in the three surveys: Belém, Brasília, Florianópolis, Goiânia, Manaus, Porto Alegre, Recife, Rio de Janeiro, Salvador and São Paulo. Analyses were performed using Software R (The R project www.r-project.org).

## Results

A total of 16,667 MSM completed the survey: 5065 (30.4%) in 2016, 2841 (17.0%) in 2017 and 8761 (52.6%) in 2018. Most participants were from the Southeast (10,418; 62.5%), followed by Northeast (2320; 13.9%), Central-west (1694; 10.2%), South (1505; 9.0%) and North (730; 4.4%). The two largest Brazilian cities had the highest number of responders: São Paulo (34.6%) and Rio de Janeiro (19.0%) (Table 1). Considering the estimated MSM population of each city, Florianópolis was the city with the greatest proportion of responders (6.6%), followed by São Paulo (4.2%) and Rio de Janeiro (4.1%).

**Table 1:** Characteristics of MSM who completed the online surveys in each Brazilian city. 2016–2018

	N (%)	Male pop. (≥18 years)^a^	Estimated MSM pop. (≥18 years)^b^	% resp.^c^	Age (years)					Race			Income (minimum wage)				Schooling (years)			
					Median (IQR)	18–24	25–29	30–34	35+	White/Asian	Black	Pardo/Native	≤1	> 1–3	> 3–10	> 10	< 10	10–12	13–16	> 16
North																				
Manaus	297 (1.8)	569,834	19,944	1.5	25 (22–30)	138 (46.5)	78 (26.3)	37 (12.5)	44 (14.8)	98 (33.8)	30 (10.3)	162 (55.9)	57 (19.2)	126 (42.4)	85 (28.6)	29 (9.8)	14 (4.8)	145 (49.5)	67 (22.9)	67 (22.9)
Belém	290 (1.7)	457,322	16,006	1.8	26 (22–32)	125 (43.1)	64 (22.1)	51 (17.6)	50 (17.2)	112 (39.0)	43 (15.0)	132 (46.0)	35 (12.1)	103 (35.5)	111 (38.3)	41 (14.1)	7 (2.4)	116 (40.3)	101 (35.1)	64 (22.2)
Porto Velho	37 (0.2)	146,727	5135	0.7	25 (22–30)	17 (45.9)	10 (27.0)	7 (18.9)	3 (8.1)	16 (43.2)	3 (8.1)	18 (48.6)	4 (10.8)	12 (32.4)	18 (48.6)	3 (8.1)	1 (2.7)	13 (37.1)	13 (37.1)	8 (22.9)
Palmas	34 (0.2)	75,543	2644	1.3	27.5 (24–32.7)	11 (32.4)	9 (26.5)	6 (17.6)	8 (23.5)	15 (44.1)	5 (14.7)	14 (41.2)	4 (11.8)	9 (26.5)	18 (52.9)	3 (8.8)	0 (0.0)	12 (35.3)	11 (32.4)	11 (32.4)
Boa Vista	31 (0.2)	89,030	3116	1.0	30 (24.5–37.5)	8 (25.8)	6 (19.4)	8 (25.8)	9 (29.0)	5 (16.1)	2 (6.5)	24 (77.4)	3 (9.7)	12 (38.7)	12 (38.7)	4 (12.9)	0 (0.0)	8 (25.8)	13 (41.9)	10 (32.3)
Rio Branco	23 (0.1)	103,808	3633	0.6	28 (24.5–32)	6 (26.1)	9 (39.1)	5 (21.7)	3 (13.0)	7 (30.4)	2 (8.7)	14 (60.9)	5 (21.7)	6 (26.1)	9 (39.1)	3 (13.0)	0 (0.0)	8 (34.8)	9 (39.1)	6 (26.1)
Macapá	18 (0.1)	120,180	4206	0.4	27 (22.5–30.7)	7 (38.9)	5 (27.8)	3 (16.7)	3 (16.7)	9 (50.0)	3 (16.7)	6 (33.3)	3 (16.7)	8 (44.4)	4 (22.2)	3 (16.7)	1 (5.6)	4 (23.5)	8 (47.1)	4 (23.5)
Northeast																				
Recife	636 (3.8)	508,405	17,794	3.6	29 (24–36)	183 (28.8)	160 (25.2)	113 (17.8)	180 (28.3)	283 (45.4)	87 (13.9)	254 (40.7)	82 (12.9)	239 (37.6)	223 (35.1)	92 (14.5)	17 (2.7)	232 (36.7)	207 (32.8)	176 (27.8)
Salvador	628 (3.8)	904,397	31,654	2.0	29 (24–35)	168 (26.8)	162 (25.8)	120 (19.1)	178 (28.3)	182 (29.4)	190 (30.7)	247 (39.9)	83 (13.2)	224 (35.7)	216 (34.4)	105 (16.7)	16 (2.6)	213 (34.0)	230 (36.7)	168 (26.8)
Fortaleza	418 (2.5)	800,792	28,028	1.5	28 (23–34)	131 (31.3)	115 (27.5)	80 (19.1)	92 (22.0)	157 (38.2)	55 (13.4)	199 (48.4)	69 (16.5)	160 (38.3)	145 (34.7)	44 (10.5)	7 (1.7)	170 (41.6)	139 (34.0)	93 (22.7)
Natal	171 (1.0)	268,193	9387	1.8	28 (24–34)	48 (28.1)	51 (29.8)	31 (18.1)	41 (24.0)	91 (53.5)	22 (12.9)	57 (33.5)	13 (7.6)	68 (39.8)	62 (36.3)	28 (16.4)	6 (3.5)	61 (35.9)	55 (32.4)	48 (28.2)
Joao Pessoa	115 (0.7)	237,865	8325	1.4	29 (24–34)	31 (27.0)	27 (23.5)	29 (25.2)	28 (24.3)	44 (38.3)	12 (10.4)	59 (51.3)	18 (15.7)	47 (40.9)	37 (32.2)	13 (11.3)	4 (3.5)	35 (30.4)	52 (45.2)	24 (20.9)
Aracaju	108 (0.6)	185,953	6508	1.7	25 (21–29)	46 (42.6)	36 (33.3)	10 (9.3)	16 (14.8)	48 (45.7)	17 (16.2)	40 (38.1)	19 (17.6)	53 (49.1)	26 (24.1)	10 (9.3)	1 (0.9)	51 (47.2)	34 (31.5)	22 (20.4)
Teresina	100 (0.6)	262,763	9197	1.1	27 (22–31.2)	39 (39.0)	25 (25.0)	18 (18.0)	18 (18.0)	27 (27.3)	18 (18.2)	54 (54.5)	20 (20.0)	37 (37.0)	38 (38.0)	5 (5.0)	2 (2.0)	34 (35.4)	33 (34.4)	27 (28.1)
Maceió	91 (0.5)	292,718	10,245	0.9	27 (23–33)	37 (40.7)	20 (22.0)	15 (16.5)	19 (20.9)	33 (36.7)	17 (18.9)	40 (44.4)	14 (15.4)	28 (30.8)	36 (39.6)	13 (14.3)	1 (1.1)	35 (38.9)	29 (32.2)	25 (27.8)
São Luís	53 (0.3)	326,863	11,440	0.5	25 (23–30)	26 (49.1)	10 (18.9)	7 (13.2)	10 (18.9)	20 (37.7)	11 (20.8)	22 (41.5)	5 (9.4)	28 (52.8)	17 (32.1)	3 (5.7)	1 (1.9)	24 (45.3)	17 (32.1)	11 (20.8)
Central-west																				
Brasília	890 (5.3)	855,306	29,936	3.0	30 (25–38)	219 (24.6)	209 (23.5)	170 (19.1)	292 (32.8)	460 (52.8)	88 (10.1)	324 (37.2)	46 (5.2)	199 (22.4)	341 (38.3)	304 (34.2)	12 (1.4)	261 (29.4)	293 (33.0)	322 (36.3)
Goiânia	570 (3.4)	450,221	15,758	3.6	28 (23–33)	169 (29.6)	174 (30.5)	113 (19.8)	114 (20.0)	287 (51.0)	61 (10.8)	215 (38.2)	64 (11.2)	201 (35.3)	227 (39.8)	78 (13.7)	32 (5.7)	222 (39.2)	167 (29.5)	146 (25.7)
Cuiabá	119 (0.7)	190,291	6660	1.8	27 (23–33)	43 (36.1)	34 (28.6)	20 (16.8)	22 (18.5)	42 (35.9)	30 (25.6)	45 (38.5)	9 (7.6)	46 (38.7)	48 (40.3)	16 (13.4)	4 (3.4)	39 (33.6)	44 (37.9)	29 (25.0)
Campo Grande	115 (0.7)	270,346	9462	1.2	28 (23–33)	42 (36.5)	28 (24.3)	24 (20.9)	21 (18.3)	58 (50.9)	19 (16.7)	37 (32.5)	8 (7.0)	41 (35.7)	50 (43.5)	16 (13.9)	4 (3.5)	45 (39.5)	36 (31.6)	29 (25.4)
Southeast																				
São Paulo	5764 (34.6)	3,889,290	136,125	4.2	30 (25–36)	1393 (24.2)	1382 (24.0)	1187 (20.6)	1801 (31.3)	3885 (68.1)	476 (8.3)	1342 (23.5)	396 (6.9)	1596 (27.7)	2566 (44.5)	1206 (20.9)	214 (3.7)	1966 (34.2)	2147 (37.4)	1421 (24.7)
Rio de Janeiro	3166 (19.0)	2,198,018	76,931	4.1	30 (24–37)	803 (25.4)	757 (23.9)	602 (19.0)	1004 (31.7)	1791 (57.1)	415 (13.2)	931 (29.7)	293 (9.3)	996 (31.5)	1285 (40.6)	592 (18.7)	150 (4.8)	1293 (41.0)	878 (27.8)	836 (26.5)
Belo Horizonte	967 (5.8)	831,360	29,098	3.3	29 (24–35)	260 (26.9)	262 (27.1)	176 (18.2)	269 (27.8)	468 (48.8)	138 (14.4)	353 (36.8)	97 (10.0)	328 (33.9)	429 (44.4)	113 (11.7)	42 (4.4)	361 (37.6)	309 (32.2)	248 (25.8)
Campinas	260 (1.6)	389,948	13,648	1.9	29 (25–38)	60 (23.1)	72 (27.7)	48 (18.5)	80 (30.8)	165 (63.7)	25 (9.7)	69 (26.6)	19 (7.3)	82 (31.5)	129 (49.6)	30 (11.5)	16 (6.2)	99 (38.1)	75 (28.8)	70 (26.9)
Vitória	160 (1.0)	114,835	4019	4.0	29 (24.7–34)	40 (25.0)	43 (26.9)	43 (26.9)	34 (21.3)	76 (48.1)	20 (12.7)	62 (39.2)	18 (11.3)	67 (41.9)	64 (40.0)	11 (6.9)	4 (2.6)	60 (37.7)	55 (34.6)	40 (25.2)
Santos	101 (0.6)	148,026	5181	1.9	31 (26–40)	16 (15.8)	26 (25.7)	24 (23.8)	35 (34.7)	68 (67.3)	7 (6.9)	26 (25.7)	7 (6.9)	29 (28.7)	50 (49.5)	15 (14.9)	2 (2.0)	27 (26.7)	46 (45.5)	26 (25.7)
South																				
Porto Alegre	648 (3.9)	489,677	17,139	3.8	30 (25–37)	150 (23.1)	149 (23.0)	134 (20.7)	215 (33.2)	526 (82.0)	30 (4.7)	85 (13.3)	29 (4.5)	196 (30.2)	303 (46.3)	120 (18.5)	20 (3.1)	242 (37.5)	203 (31.4)	181 (28.0)
Curitiba	499 (3.0)	615,841	21,554	2.3	30 (25–36)	122 (24.4)	118 (23.6)	115 (23.0)	144 (28.9)	347 (70.4)	32 (6.5)	114 (23.1)	28 (5.6)	147 (29.5)	248 (49.7)	76 (15.2)	16 (3.2)	169 (34.0)	161 (32.4)	151 (30.4)
Florianópolis	358 (2.1)	155,364	5438	6.6	29 (25–34)	88 (24.6)	100 (27.9)	85 (23.7)	85 (23.7)	259 (73.2)	26 (7.3)	69 (19.5)	15 (4.2)	132 (36.9)	153 (42.7)	58 (16.2)	18 (5.0)	147 (41.1)	107 (29.9)	86 (24.0)
Total	16,667 (100.0)	15,948,916	558,212	3.0	29 (24–36)	4426 (26.6)	4141 (24.8)	3281 (19.7)	4818 (28.9)	9579 (58.1)	1884 (11.4)	5014 (30.4)	1463 (8.7)	5220 (31.3)	6950 (41.7)	3034 (18.2)	612 (3.7)	6092 (36.7)	5539 (33.4)	4349 (26.2)

Missing (participant did not want to answer or did not know): age = 1; race = 190; schooling = 75; sexual orientation = 86; steady partner = 76; test lifetime = 51.

^a^According to Brazilian CENSO 2010 (https://sidra.ibge.gov.br/tabela/1209)

^b^Considering the proportion of MSM among Brazilian male population between 15 and 64 years (3.5%) (http://www.aids.gov.br/pt-br/pub/2016/pesquisa-de-conhecimentos-atitudes-e-praticas-na-populacao-brasileira-pcap-2013)

^c^Proportion of MSM who completed the web-based surveys in relation to estimated MSM population

Overall, median age was 29 years (IQR: 24–36). The age distribution of the participants was shifted towards younger MSM (18–24 years) in most of cities in the North, Northeast and Central-west regions, representing almost half of responders from Manaus (46.5%) and Belém (43.1%), the two largest urban areas in the North. In large cities from the Southeast, South and Brasília the proportion of older MSM (35+ years) was higher (31% in São Paulo and Rio de Janeiro). Most responders from the North and Northeast self-declared pardo or native, and Salvador was the city with the greatest proportion of black MSM (30.7%). Conversely, more than half of responders from the Central-west, Southeast and South self-reported as white, except in Cuiabá (35.9%). Southern cities had the greatest proportion of white MSM (highest in Porto Alegre with 82.0%), followed by the cities from São Paulo State (68.1% in São Paulo city). Family monthly income was shifted towards lower income in cities in the North and Northeast with approximately 20% of responders earning one minimum wage in Manaus (19.2%), Rio Branco (21.7%), Aracaju (17.6%), and Teresina (20%). Most responders from the Central-west, Southeast and South regions had middle income (> 3 to 10 minimum wages), and the greatest proportion of MSM with higher income (> 10 minimum wages) was observed in Brasília (34.2%), São Paulo (20.9%) and Rio de Janeiro (18.7%). Having > 12 years of schooling (equivalent to high school) was reported by more than half of the respondents in every city except for Manaus (45.8%). Brasilia was the city with the greatest proportion of MSM with > 16 years of schooling (36.3%).

Most participants self-declared as gay or homosexual (89.9%) (Table 2). The proportion of MSM with a steady partner varied across the country, ranging in the largest urban areas from 19.5% in Belém to 28.2% in Brasília. Most MSM reported binge drinking (70.2%) with only slight variability by region and use of stimulants was more prevalent in the South and Southeast (~ 20%), the highest proportions were observed in the cities from São Paulo state (24.1% in São Paulo city), Brasília (23.1%) and Florianópolis (22.9%). Overall, 13.1% of participants reported an STI, Florianópolis (18.6%) had the highest proportion among the cities with > 300 responders.

**Table 2:** Sexual orientation, steady partner and behaviors of MSM who completed the online surveys in each Brazilian city. 2016–2018

	Total	Sexual Orientation (Gay or homosexual)	Steady partner	Binge drinking^a^	Use of Stimulants ^a, b^	STI^a, c^	HIV testing (never)	Sex with > 5 men^a^	Condomless receptive anal sex^a^	The HIV Incidence Risk Index for MSM (HIRI-MSM)^d^		HIV perceived risk^e^		
										Low Risk	High risk	No risk	Low risk	High risk
North														
Manaus	297	242 (83.2)	72 (24.2)	191 (64.3)	30 (10.1)	32 (10.9)	75 (25.5)	114 (38.4)	118 (39.9)	120 (40.4)	177 (59.6)	59 (21.5)	134 (48.9)	81 (29.6)
Belém	290	240 (83.9)	56 (19.5)	203 (70.0)	31 (10.7)	32 (11.1)	76 (26.5)	105 (36.2)	122 (42.2)	116 (40.0)	174 (60.0)	64 (23.5)	144 (52.9)	64 (23.5)
Porto Velho	37	31 (83.8)	6 (16.7)	28 (75.7)	5 (13.5)	4 (10.8)	7 (19.4)	13 (35.1)	19 (52.8)	9 (24.3)	28 (75.7)	3 (8.6)	19 (54.3)	13 (37.1)
Palmas	34	29 (85.3)	9 (26.5)	25 (73.5)	3 (8.8)	6 (17.6)	5 (15.2)	9 (26.5)	17 (50.0)	11 (32.4)	23 (67.6)	4 (11.8)	16 (47.1)	14 (41.2)
Boa Vista	31	27 (87.1)	10 (32.3)	22 (71.0)	6 (19.4)	6 (19.4)	1 (3.2)	12 (38.7)	13 (41.9)	12 (38.7)	19 (61.3)	7 (22.6)	18 (58.1)	6 (19.4)
Rio Branco	23	23 (100.0)	7 (31.8)	18 (78.3)	3 (13.0)	7 (30.4)	3 (13.0)	11 (47.8)	10 (43.5)	8 (34.8)	15 (65.2)	3 (13.0)	15 (65.2)	5 (21.7)
Macapá	18	13 (72.2)	6 (33.3)	10 (55.6)	3 (16.7)	3 (17.6)	3 (16.7)	7 (38.9)	9 (50.0)	7 (38.9)	11 (61.1)	3 (16.7)	6 (33.3)	9 (50.0)
Northeast														
Recife	636	550 (87.0)	173 (27.3)	437 (68.8)	81 (12.7)	64 (10.2)	130 (20.4)	251 (39.5)	255 (40.3)	253 (39.8)	383 (60.2)	121 (20.2)	329 (54.9)	149 (24.9)
Salvador	628	555 (88.8)	147 (23.6)	451 (71.8)	113 (18.0)	73 (11.8)	106 (17.0)	277 (44.1)	251 (40.2)	241 (38.4)	387 (61.6)	119 (20.2)	312 (53.1)	157 (26.7)
Fortaleza	418	391 (93.8)	94 (22.7)	288 (68.9)	56 (13.4)	46 (11.2)	103 (25.0)	151 (36.1)	158 (38.3)	173 (41.4)	245 (58.6)	70 (17.6)	214 (53.8)	114 (28.6)
Natal	171	155 (91.2)	33 (19.5)	118 (69.0)	19 (11.1)	23 (13.5)	43 (25.3)	66 (38.6)	76 (45.0)	67 (39.2)	104 (60.8)	22 (13.2)	96 (57.5)	49 (29.3)
Joao Pessoa	115	106 (93.0)	30 (26.1)	75 (65.2)	17 (14.8)	14 (12.3)	18 (15.8)	52 (45.2)	44 (38.3)	46 (40.0)	69 (60.0)	16 (14.7)	55 (50.5)	38 (34.9)
Aracaju	108	93 (86.1)	20 (18.7)	77 (71.3)	12 (11.1)	10 (9.3)	36 (33.6)	34 (31.5)	39 (36.4)	49 (45.4)	59 (54.6)	24 (23.3)	53 (51.5)	26 (25.2)
Teresina	100	84 (85.7)	25 (26.0)	63 (63.0)	6 (6.0)	9 (9.4)	26 (27.1)	35 (35.0)	37 (37.4)	43 (43.0)	57 (57.0)	18 (18.9)	42 (44.2)	35 (36.8)
Maceió	91	79 (86.8)	26 (29.2)	65 (71.4)	11 (12.1)	6 (6.9)	20 (22.2)	33 (36.3)	35 (38.5)	35 (38.5)	56 (61.5)	15 (17.9)	54 (64.3)	15 (17.9)
São Luís	53	50 (94.3)	10 (18.9)	33 (62.3)	5 (9.4)	9 (17.0)	18 (34.0)	21 (39.6)	22 (41.5)	21 (39.6)	32 (60.4)	9 (19.1)	22 (46.8)	16 (34.0)
Central-west														
Brasília	890	795 (89.6)	250 (28.2)	632 (71.1)	206 (23.1)	110 (12.5)	113 (12.7)	399 (44.8)	359 (40.5)	322 (36.2)	568 (63.8)	133 (15.8)	481 (57)	230 (27.3)
Goiânia	570	509 (89.9)	123 (21.7)	405 (71.3)	103 (18.1)	89 (16.1)	126 (22.1)	230 (40.4)	227 (40.0)	213 (37.4)	357 (62.6)	104 (19.7)	267 (50.7)	156 (29.6)
Cuiabá	119	102 (86.4)	22 (18.6)	84 (70.6)	19 (16.0)	19 (16.4)	22 (18.6)	52 (43.7)	53 (45.3)	36 (30.3)	83 (69.7)	15 (13.5)	56 (50.5)	40 (36.0)
Campo Grande	115	98 (85.2)	19 (16.5)	80 (69.6)	25 (21.7)	20 (17.9)	22 (19.1)	44 (38.3)	50 (44.2)	40 (34.8)	75 (65.2)	16 (14.3)	60 (53.6)	36 (32.1)
Southeast														
São Paulo	5764	5201 (90.6)	1341 (23.3)	4005 (69.6)	1421 (24.7)	763 (13.4)	790 (13.7)	2664 (46.2)	2375 (41.4)	1951 (33.8)	3813 (66.2)	911 (16.8)	3033 (55.9)	1484 (27.3)
Rio de Janeiro	3166	2820 (89.6)	775 (24.6)	2287 (72.3)	624 (19.8)	398 (12.7)	541 (17.1)	1438 (45.4)	1331 (42.1)	1104 (34.9)	2062 (65.1)	499 (16.9)	1640 (55.6)	810 (27.5)
Belo Horizonte	967	881 (91.3)	227 (23.6)	693 (71.7)	180 (18.6)	123 (13.0)	177 (18.3)	386 (39.9)	392 (40.9)	362 (37.4)	605 (62.6)	134 (14.5)	504 (54.7)	283 (30.7)
Campinas	260	235 (91.1)	56 (21.6)	173 (66.5)	59 (22.7)	31 (12.1)	47 (18.1)	97 (37.3)	115 (45.3)	102 (39.2)	158 (60.8)	35 (14.2)	137 (55.5)	75 (30.4)
Vitória	160	148 (94.9)	34 (21.5)	115 (71.9)	36 (22.5)	21 (13.4)	22 (13.8)	58 (36.2)	72 (46.2)	44 (27.5)	116 (72.5)	22 (14.9)	83 (56.1)	43 (29.1)
Santos	101	92 (91.1)	28 (27.7)	68 (67.3)	14 (13.9)	16 (16.0)	12 (11.9)	38 (37.6)	39 (39.0)	41 (40.6)	60 (59.4)	21 (22.1)	46 (48.4)	28 (29.5)
South														
Porto Alegre	648	555 (88.8)	167 (25.9)	441 (68.2)	129 (20.0)	77 (12.1)	93 (14.4)	295 (45.5)	274 (42.3)	238 (36.7)	410 (63.3)	89 (14.6)	361 (59.2)	160 (26.2)
Curitiba	499	455 (91.5)	110 (22.2)	335 (67.1)	102 (20.4)	68 (13.9)	63 (12.7)	217 (43.5)	197 (39.6)	170 (34.1)	329 (65.9)	70 (14.8)	255 (53.9)	148 (31.3)
Florianópolis	358	324 (90.8)	79 (22.1)	262 (73.2)	82 (22.9)	66 (18.6)	56 (15.7)	160 (44.7)	156 (43.7)	111 (31.0)	247 (69.0)	59 (17.4)	177 (52.2)	103 (30.4)
Total	16,667	14,909 (89.9)	3955 (23.8)	11,684 (70.2)	3401 (20.4)	2145 (13.1)	2754 (16.6)	7269 (43.6)	6865 (41.4)	5945 (35.7)	10,722 (64.3)	2665 (17.0)	8629 (55.0)	4387 (28.0)

^a^During the previous 6 months

^b^Cocaine, poppers, crack, or amphetamines

^c^Syphilis, gonorrhea, or rectal chlamydia

^d^The HIRI-MSM was calculated based on sexual behavior in the previous 6 months (number of partners, condomless receptive anal intercourse, sex with HIV-positive partner), age and use of stimulants, being stratified in low risk (< 10 points) and high risk (≥10 points; PrEP is recommended). “Unknown” answers scored 0 points on the HIRI-MSM

^e^In the next 12 months

**Fig. 1:**
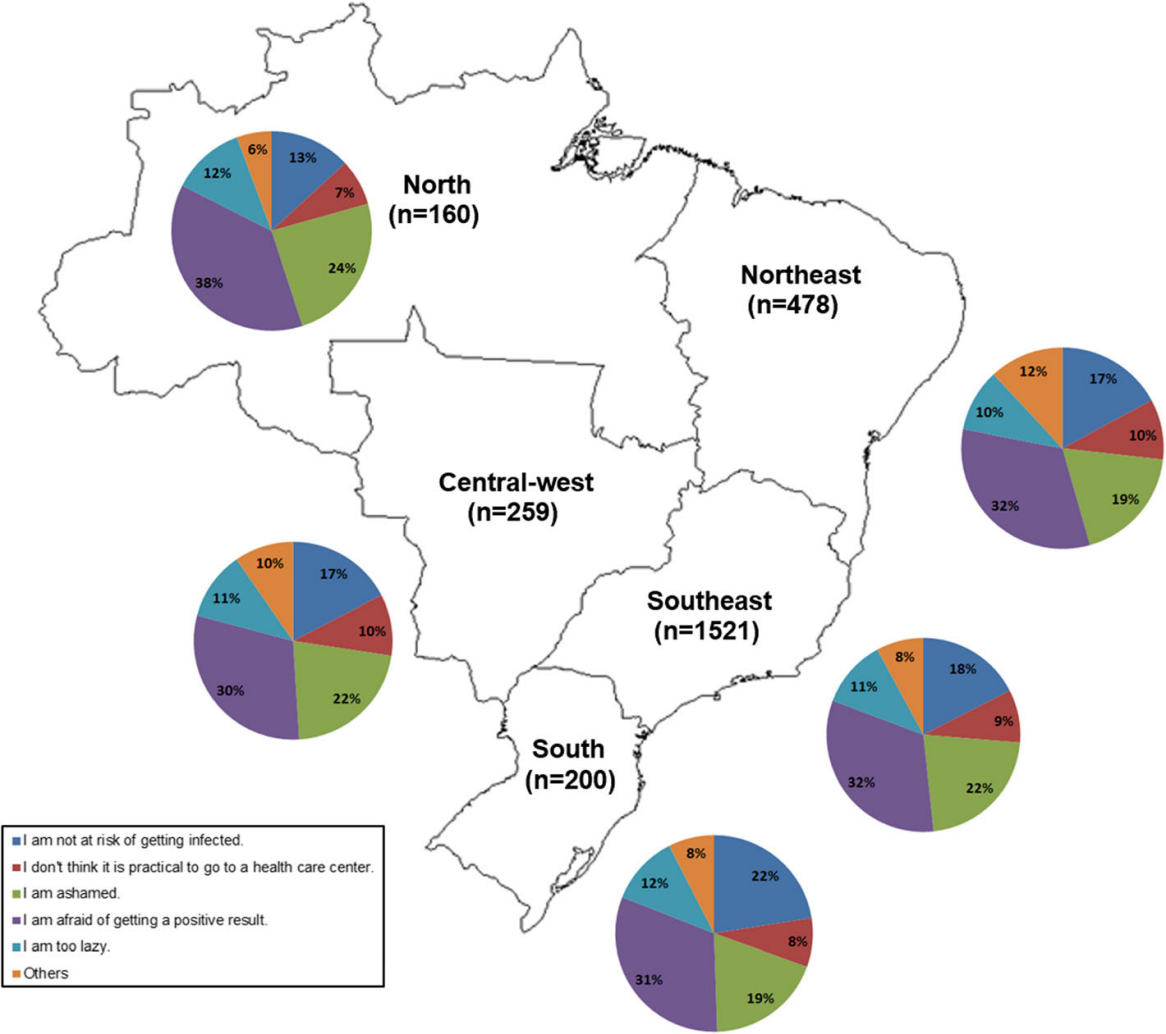
Distribution of responses to the question “Why have you never tested for HIV?” by Brazilian Geopolitical Region (*n* = 2618)

The proportion of MSM who reported never having tested for HIV was higher in the North and Northeast when compared to other regions: 26% in Manaus and Belém and 12% in Santos, Curitiba and Brasília. The main reason for never testing for HIV were, in order, “I am afraid of getting a positive result” (851; 32.4%), “I am ashamed” (559; 21.3%), “I am not at risk of getting infected” (459; 17.5%), “I am too lazy” (293; 11.2%), “I don’t think it is practical to go to a health care center” (234; 8.9%) and others (227; 8.7%). This pattern was observed in all Brazilian regions except for the South where the response “I am not at risk of getting infected” was more frequent than “I am ashamed” (22% vs. 19%). In the North, more MSM reported “I am afraid of getting a positive result” (38%) in comparison to the other regions (Fig. 1).

Overall, the number of responders who reported sex with more than five men was lower in Northern and Northeastern cities compared to the other regions, around 35%. In contrast, this proportion was higher in Southeastern cities, São Paulo (46.2%), Rio de Janeiro (45.4%), Brasília (44.8%) and in the Southern cities, ranging from 43.5% in Curitiba to 45.5% in Porto Alegre. Condomless receptive anal sex was reported by 6865 participants (41.4%) with little heterogeneity by city. Most participants met criteria for “high risk” according to the HIRI-MSM (64.3%), indicating that PrEP should be recommended. Conversely, only 28.0% of participants reported high HIV perceived risk, and this proportion was lower in Belém (23.5%) and Recife (24.9%).

Table 3 shows that irrespective of the definition of high-risk behavior, overall, high-risk behavior was associated with a 3-fold increase in the odds of high HIV perceived risk. That said, less than a half of those with high-risk behavior (as measured by a score > 10 in the HIRI-MSM, condomless receptive anal sex, or > 5 partners in the prior 6 months) perceived themselves at high risk. In contrast, among those reporting low-risk behavior, around one-fifth perceived themselves at high-risk (13.9% as measured by the HIRI-MSM, 19.7% who report condomless anal sex and 18.8% who had ≤5 partners). Finally, we found evidence for a significant association between sexual behavior with HIV perceived risk in the 11 cities according to the HIRI-MSM and report of condomless anal sex (Tables 4 and 5).

**Table 3:** Association between HIV risk behavior and HIV perceived risk among MSM who completed the online surveys in Brazil

	HIV Perceived risk^a^		High HIV perceived risk OR (95% CI)
	Low	High	
The HIV incidence risk index for MSM^b^			
Low	4860 (86.1)	784 (13.9)	Ref.
High	6434 (64.1)	3603 (35.9)	3.47 (3.19–3.78)
Condomless receptive anal sex^c^			
No	7400 (80.3)	1811 (19.7)	Ref.
Yes	3847 (60.1)	2552 (39.9)	2.71 (2.52–2.91)
Number of male partners^c^			
≤ 5	7213 (81.2)	1671 (18.8)	Ref.
> 5	4081 (60.0)	2716 (40.0)	2.87 (2.67–3.09)

^a^In the next 12 months

^b^The HIRI-MSM was calculated based on sexual behavior in the previous 6 months (number of partners, condomless receptive anal intercourse, sex with HIV-positive partner), age and use of stimulants, being stratified in low risk (< 10 points) and high risk (≥10 points; PrEP is recommended).“Unknown” answers scored 0 points on the HIRI-MSM

^c^During the previous 6 months

**Table 4:** HIV risk behavior vs. HIV perceived risk among MSM from 11 cities in Brazil

	HIV risk behavior^a^	HIV perceived risk ^b^		*p*-value
		Low	High	
North				
Manaus	Low	99(85.3)	17(14.7)	<.001
	High	94(59.5)	64(40.5)	
Belém	Low	102(89.5)	12(10.5)	<.001
	High	106(67.1)	52(32.9)	
Northeast				
Recife	Low	203(84.9)	36(15.1)	<.001
	High	247(68.6)	113(31.4)	
Salvador	Low	198(85.3)	34(14.7)	<.001
	High	233(65.4)	123(34.6)	
Central-west				
Brasília	Low	268(86.7)	41(13.3)	<.001
	High	346(64.7)	189(35.3)	
Goiânia	Low	166(83.4)	33(16.6)	<.001
	High	205(62.5)	123(37.5)	
Southeast				
São Paulo	Low	1604(86.9)	241(13.1)	<.001
	High	2340(65.3)	1243(34.7)	
Rio de Janeiro	Low	910(87.8)	126(12.2)	<.001
	High	1229(64.2)	684(35.8)	
Belo Horizonte	Low	287(83.2)	58(16.8)	<.001
	High	351(60.9)	225(39.1)	
South				
Porto Alegre	Low	198(87.2)	29(12.8)	<.001
	High	252(65.8)	131(34.2)	
Curitiba	Low	137(84.0)	26(16.0)	<.001
	High	188(60.6)	122(39.4)	
Brazil	Low	4860 (86.1)	784 (13.9)	<.001
	High	6434 (64.1)	3603 (35.9)	

^a^measured by the HIRI-MSM, which was calculated based on sexual behavior in the previous 6 months (number of partners, condomless receptive anal intercourse, sex with HIV-positive partner), age and use of stimulants, being stratified in low risk (< 10 points) and high risk (≥10 points; PrEP is recommended)

^b^HIV perceived risk (in the next 12 months) was dichotomized in low (no/low) and high risk

**Table 5:** Condomless receptive anal sex vs. HIV perceived risk among MSM from 11 cities in Brazil

	Condomless receptive anal sex^a^	HIV perceived risk ^b^		*p*-value
		Low	High	
North				
Manaus	No	133(79.6)	34(20.4)	<.001
	Yes	60(56.6)	46(43.4)	
Belém	No	137(85.6)	23(14.4)	<.001
	Yes	71(64.0)	40(36.0)	
Northeast				
Recife	No	295(82.4)	63(17.6)	<.001
	Yes	153(64.3)	85(35.7)	
Salvador	No	292(81.3)	67(18.7)	<.001
	Yes	138(60.8)	89(39.2)	
Central-west				
Brasília	No	400(79.4)	104(20.6)	<.001
	Yes	212(62.9)	125(37.1)	
Goiânia	No	247(77.9)	70(22.1)	<.001
	Yes	123(59.1)	85(40.9)	
Southeast				
São Paulo	No	2592(81.5)	590(18.5)	<.001
	Yes	1334(60.0)	889(40.0)	
Rio de Janeiro	No	1397(81.6)	314(18.4)	<.001
	Yes	741(60.0)	494(40.0)	
Belo Horizonte	No	414(76.4)	128(23.6)	<.001
	Yes	218(58.6)	154(41.4)	
South				
Porto Alegre	No	290(81.5)	66(18.5)	<.001
	Yes	160(63.2)	93(36.8)	
Curitiba	No	221(76.5)	68(23.5)	<.001
	Yes	103(56.3)	80(43.7)	
Brazil	No	7400 (80.3)	1811 (19.7)	<.001
	Yes	3847 (60.1)	2552 (39.9)	

^a^During the previous 6 months

^b^HIV perceived risk (in the next 12 months) was dichotomized in low (no/low) and high risk

Overall, the association of socio-demographic and behavioral characteristics with high-risk behavior as measured by the HIRI-MSM was consistent across the cities (Table 6). Younger age, being gay or homosexual, having a steady partner, binge drinking, STI diagnosis and ever testing for HIV increased the odds of high-risk behavior. As participants from São Paulo and Rio de Janeiro make up the majority of the study population, the results of Brazil as a whole greatly reflect those for these two cities. One notable exception was the association of having a steady partner increasing the odds of high-risk behavior which is most strongly observed in Goiania. Similarly, the association of binge drinking with high-risk behavior is more pronounced in the North, Central-west, and South, and not apparent in the Northeast.

**Table 6:** Association, measured by the adjusted odds ratios (aOR, 95% confidence interval) between MSM characteristics and high-risk behavior as measured by the HIV Incidence Risk Index for MSM (HIRI-MSM), by city, 2016–2018

	Total	Young age: 18–24 years (ref: > 24 years	Black/pardo/native (ref: white/Asian)	≤ 3 MW income (ref: > 3 MW)	≤ 12 years of Schooling (ref: > 12 years)	Gay or homosexual (ref: Other than gay)	Steady partner (ref: no)	Binge drinking^a^ (ref: no)	STI^a, c^ (ref: no)	Ever HIV testing (ref: never)
North										
Manaus	297	1.20(0.67–2.18)	1.48(0.86–2.55)	0.78(0.44–1.37)	1.13(0.65–2.00)	0.95(0.47–1.89)	1.54(0.84–2.88)	**2.15(1.27–3.64)**	1.48(0.63–3.69)	**2.44(1.36–4.42)**
Belém	290	1.65(0.91–3.03)	1.32(0.77–2.28)	0.62(0.35–1.09)	1.11(0.60–2.02)	1.42(0.69–2.92)	0.71(0.37–1.39)	**2.76(1.58–4.90)**	**5.35(1.93–19.12)**	0.62(0.33–1.16)
Northeast										
Recife	636	**1.71(1.12–2.62)**	0.81(0.57–1.13)	0.73(0.51–1.05)	1.15(0.78–1.71)	**1.67(1.03–2.72)**	1.19(0.82–1.73)	1.18(0.82–1.68)	1.32(0.76–2.35)	1.14(0.74–1.75)
Salvador	628	1.12(0.72–1.73)	0.83(0.56–1.22)	0.95(0.65–1.39)	1.40(0.90–2.17)	**2.59(1.52–4.51)**	1.11(0.74–1.67)	1.33(0.92–1.94)	**3.82(2.01–7.93)**	1.61(1.00–2.59)
Central-west										
Brasília	890	1.29(0.87–1.91)	1.05(0.78–1.42)	0.86(0.60–1.24)	0.83(0.58–1.21)	**2.46(1.55–3.93)**	1.20(0.86–1.67)	**1.84(1.34–2.53)**	**3.28(1.96–5.80)**	1.09(0.69–1.72)
Goiânia	570	1.39(0.88–2.21)	0.93(0.64–1.35)	0.98(0.65–1.46)	**1.55(1.02–2.38)**	1.28(0.70–2.31)	**1.72(1.10–2.74)**	**2.00(1.34–3.00)**	**2.37(1.38–4.23)**	1.34(0.84–2.14)
Southeast										
São Paulo	5764	**1.46(1.25–1.71)**	1.04(0.92–1.18)	0.90(0.79–1.03)	0.96(0.84–1.11)	**2.05(1.70–2.48)**	1.04(0.90–1.19)	**2.08(1.84–2.34)**	**2.48(2.04–3.04)**	**1.42(2.04–3.04)**
Rio de Janeiro	3166	**1.40(1.14–1.72)**	0.94(0.80–1.10)	1.09(0.92–1.30)	0.97(0.81–1.16)	**1.73(1.36–2.20)**	1.16(0.97–1.39)	**1.69(1.43–2.00)**	**2.60(1.98–3.46)**	**1.75(1.42–2.16)**
Belo Horizonte	967	0.93(0.67–1.31)	1.00(0.75–1.32)	1.18(0.88–1.59)	0.93(0.68–1.28)	**2.17(1.35–3.51)**	1.02(0.74–1.42)	**1.74(1.29–2.34)**	**1.58(1.04–2.46)**	1.09(0.76–1.57)
South										
Porto Alegre	648	1.27(0.80–2.04)	0.79(0.51–1.23)	1.11(0.76–1.63)	0.94(0.63–1.40)	**2.01(1.16–3.51)**	1.05(0.71–1.57)	**2.09(1.46–2.99)**	**2.49(1.39–4.74)**	1.21(0.73–1.98)
Curitiba	499	1.09(0.65–1.84)	1.28(0.82–2.03)	0.90(0.58–1.42)	0.86(0.54–1.37)	**2.09(1.02–4.31)**	1.16(0.71–1.91)	**2.12(1.40–3.21)**	**2.00(1.07–4.00)**	1.65(0.89–3.02)
Brazil	16,667	**1.34(1.23–1.46)**	0.97(0.91–1.04)	0.96(0.89–1.04)	1.00(0.93–1.09)	**1.91(1.71–2.13)**	**1.13(1.04–1.22)**	**1.88(1.75–2.02)**	**2.30(2.06–2.59)**	**1.45(1.32–1.59)**

The HIRI-MSM was calculated based on sexual behavior in the previous 6 months (number of partners, condomless receptive anal intercourse, sex with HIV-positive partner), age and use of stimulants, being stratified in low risk (< 10 points) and high risk (≥10 points; PrEP is recommended). “Unknown” answers scored 0 points on The HIRI-MSM

*MW* minimum wage

^a^During the previous 6 months

^b^Cocaine, poppers, crack, or amphetamines

^c^Syphilis, gonorrhea, or rectal chlamydia

all entries in bold have significance (*p*<=0.05)

**Fig. 2:**
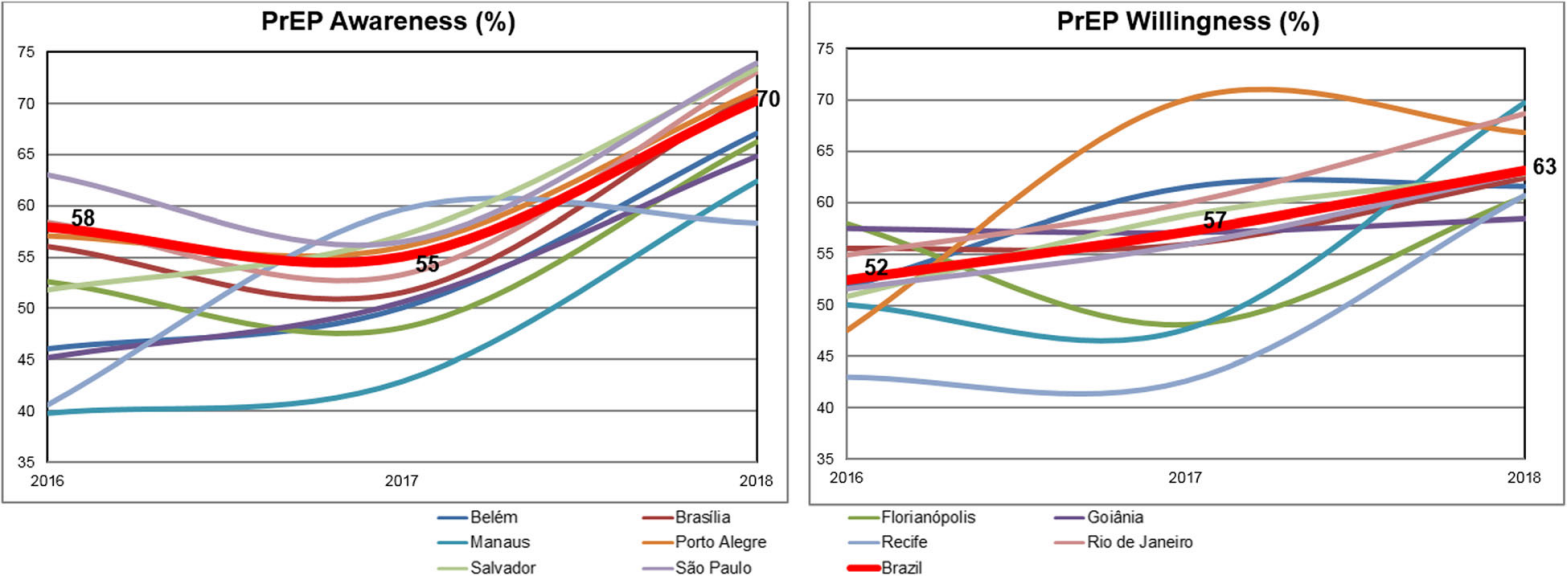
Awareness and willingness to use PrEP among MSM from 10 Brazilian State Capitals from 2016 to 2018

Overall, PrEP awareness increased overtime in Brazil from 58% in 2016 to 70% in 2018 (Fig. 2). Among the 10 cities evaluated in all surveys, São Paulo had the highest proportion of awareness in 2016 (63%) and 2018 (74%), and Recife the lowest in 2018 (58%). Manaus had the greatest increase in PrEP awareness (from 40% in 2016 to 62% in 2018). Willingness to use PrEP also increased overtime in Brazil, from 52% in 2016 to 63% in 2018. The highest increase in willingness to use PrEP was in Manaus, from the lowest value in 2016 (50%) to the highest in 2018 (70%). A substantial increase in willingness to use PrEP was also observed for Porto Alegre (48% in 2016 to 67% in 2018) and Rio de Janeiro (55% in 2016 to 69% in 2019). Across the years, willingness to use PrEP was almost stable in Goiânia (58% in 2016 and 2018), Florianópolis (58% in 2016 and 61% in 2018) and Brasília (56% in 2016 to 62% in 2018) (Fig. 2).

## Discussion

In the present, we described the socio-demographic characteristics as well as sexual behavior and HIV perceived risk by city across all regions of Brazil. We noted significant heterogeneity with respect to some factors (i.e. socio-demographic characteristics, stimulant use and never testing for HIV) and a homogeneous profile for others (binge drinking and sexual behavior). Moreover, our results highlight which factors were most associated with high-risk behavior by city and in the country.

One strength of this study was the inclusion of a large number of MSM living in Brazilian state capitals, in the Federal District and in two large cities from São Paulo state. Considering the estimated population of MSM in each city, the highest proportions of MSM completing the questionnaire were from the South, Southeast and Central-west regions. Compared to the North and Northeast, these regions are more developed likely providing greater access to and use of mobile phones and cell phone data services and/or public Wi-Fi services on a daily basis [17], thus facilitating participation. Differential participation among cities could also result from differential use of apps for sexual encounters by the MSM community of each city.

We observed similarities among MSM from 1) the South, Southeast and Brasilia versus those from 2) the North and Northeast. Different from group 1), for group 2) a greater proportion of MSM were young, non-white, of lower income and lower schooling, which are characteristics of the population most vulnerable to HIV infection in Brazil [7]. These group differences could be a reflection of the socio-demographic characteristics of the population from these regions according to national data [18]. Despite large inequalities, Brazil has a large number of internet users in all social strata: 58% of individuals receiving one minimum wage per month (~US$260.00) have access to internet [17]. According to estimates, 84% of Brazilians have a mobile phone [19] and 96% have access to internet/apps via mobile phones [20]. This supports the use of apps and social media to broadcast information on sexual education and HIV/STI treatment and prevention.

MSM reporting high-risk behavior measured by the HIRI-MSM, condomless receptive anal sex and increased number of partners were more likely to perceive themselves at higher risk, which is consistent with a web-based study conducted among 800 MSM from the United States [21]. However, we also found that a high proportion of MSM reporting high-risk behavior do not perceive themselves at high-risk for HIV (~ 60%). This difference was also observed in other studies conducted in different settings [22–30] and could reflect a dissonance between sexual behavior and HIV transmission knowledge. Previous studies have shown a relationship between sexual behavior or low HIV perceived risk and mistaken beliefs about HIV transmission and epidemiology [31, 32]. A Brazilian study conducted in 2008/9 evaluated HIV knowledge among MSM from 10 Brazilian cities and found that 41% of the sample had lower than average knowledge with participants still reporting beliefs in incorrect modes of HIV transmission such as through the use of a public restroom [33]. In another study conducted in 2016 [34], knowledge was found to vary by region with an overall prevalence of high level of knowledge of 24% that ranged from 5% in Fortaleza to 34% in São Paulo. Importantly, low HIV perceived risk may be a barrier to PrEP uptake [35]. Health care providers should take every opportunity to provide and reinforce information on HIV transmission and prevention. This would empower MSM to make decisions and manage their risk safely.

In our analysis of factors associated with high-risk behavior, we found that MSM who self-declared as gay or homosexual compared to other sexual orientations (bisexual, heterosexual or other) had greater odds of high-risk behavior. A longitudinal analysis partner-level data on self-declared gay and bisexual men’s behaviors has shown that behaviors depend on the type of relationship (casual versus serious) and on the type of condomless anal sex (receptive versus insertive) [36]. Of relevance to our findings, bisexual men reported more insertive condomless anal sex with casual male partners compared to self-identified gay men [36]. Although the proportion of non-gay MSM in the sample was low and includes self-declared bisexual MSM, our results likely reflect the higher frequency of receptive condomless anal sex among self-declared gay or homosexual as compared to men who report other sexual orientations. This finding is worrisome specially when coupled with those from a 2016 cross-sectional analysis of over 37 thousand young army conscripts in Brazil, 4.4% of which reported being MSM, who reported significant less condom use with casual partners compared to stable partners [37].

MSM who reported binge drinking were also found at higher odds of high-risk behavior. Binge drinking is known to increase high-risk behaviors among MSM [38–40]. In our study, binge drinking was highly prevalent across the cities (~ 70%), while more MSM from the Central-west, South and Southeast regions (~ 20%) reported use of stimulants (compared to ~ 10% in the North and Northeast). Alcohol is widely used, accepted and easy to access in Brazil [41]. Stimulants, in contrast, are expensive and illegal; access may be easier in larger urban areas, what may explain these disparities [42].

Regarding our socio-demographic factors, though there was no association of race, income or education with high-risk behavior (be it overall or by city except for 1 city), we found that younger age was associated with an increased odds of high-risk behavior. This finding is of great concern and might help explain the recent rise in HIV incidence among MSM aged 16–24 years in Brazil [7]. A recent Thai study reported similar results among MSM attending a gay sauna, where young MSM were at higher-risk compared to older MSM due to their higher risk behaviors and false perception of low HIV risk [43]. One explanation of this finding might be that younger individuals may be more fearless of HIV and/or optimistic of HIV treatment and prevention strategies [44–46]. In a web-based survey conducted among MSM from the US, younger MSM had higher knowledge of HIV prevention compared to the older, but no differences regarding HIV perceived risk [47]. However, in Brazil, sexual education at schools or within the families are still a taboo and may impact knowledge of HIV transmission risk and prevention strategies [33]. Young-friendly interventions to increase awareness of HIV risk behaviors and prevention technologies are urgent to stop new HIV infections among young MSM in Brazil.

Having a steady partner was also associated with increased odds of high-risk behavior. Risk perception decreases when feelings of trust grow in relationships [48]. Accordingly, MSM may feel more comfortable to have condomless sex with a steady partner and may perceive no or low risk with this behavior [49]. An open conversation between the couple or with the support of a health professional would be beneficial for risk management. PrEP could be an option in case of open or non-monogamic relationships as it does not depend on an agreement during sex (as condoms do). Moreover, when thinking specifically about serodiscordant couples, recent results from Opposites Attract suggest that PrEP might be useful in the initial months of a relationship or during the first months of ART initiation, after which ART-induced viral suppression is likely sufficient [50]. Here, the need to disseminate the knowledge that U=U (undetectable = untransmissible) should be emphasized [51]. As suggested in a recent review of HIV epidemiology in Latin America [4], U=U knowledge “empowers people living with HIV, improves adherence and decreases self-stigma” and it may also improve intimate relationships by decreasing fear of transmission.

Finally, having an STI and ever testing for HIV were both associated with increased odds of high-risk behavior. In fact, the association between having an STI with high-risk behavior was the largest in magnitude when compared to all other factors. In Brazil, STI diagnosis still relies extensively on syndromic management [52] suggesting that these infections were symptomatic. The observed association of high-risk behavior with STI diagnosis evidences the concomitant increased risk of HIV infection to which these men are exposed to. The interaction with health professionals at the time of STI diagnosis needs to be used for the provision of information on HIV risk thus increasing knowledge and awareness of one’s risk. This highlights the importance of health care providers as source of information and sexual education. Though it is comforting to know that MSM who engage in high-risk behavior were also more likely to test for HIV, the magnitude of the effect of STI diagnosis was much higher than that of ever testing. Of note is the present study’s conservative assessment of HIV testing as at least once in lifetime.

Overall, almost 17% of the surveyed MSM reported never having tested for HIV (median age 29 years), which can be considered quite high as the CDC recommends that everyone aged 13–64 years should be tested at least once and that sexually active gay and bisexual men benefit from more frequent testing (e.g. every 3 to 6 months) [53]. This proportion was even higher in the North and the Northeast. This may be related to limited access to health services in these regions, though only 7–10% of those who never tested reported that it was not practical to go to a health care center. The main reasons for not testing (“I am afraid of getting a positive result” and “I am ashamed”) reflects the persistent HIV stigma in Brazil with fear of HIV stigma hindering HIV testing. In a study conducted in New York City, MSM and transgender women afraid of HIV stigma were less likely to get tested [54, 55]. In addition, some MSM already face stigma for being gay and a possible HIV diagnosis would represent a new stigma to bear. HIV testing is a key technology within the HIV prevention package, it is the necessary step to linkage to care and treatment for those with HIV infection and to prevention services for those with a negative result. Information on the benefits of early HIV diagnosis and antiretroviral therapy initiation to decrease comorbidities related and not related to AIDS [56] and to decrease HIV transmission [50, 57] are essential to decrease stigma, increase HIV testing and, as consequence, decrease new cases of HIV. HIV self-testing, which is available commercially in Brazil, can play in an important role in increasing testing, although awareness of this technology is still low in the country [11, 12, 58]. A previous analysis verified that MSM willing to use PrEP were also willing to use HIV self-testing, indicating that both technologies could be offered in the same platform, which could be web-based [58].

In this regard, it is encouraging that awareness and willingness to use PrEP increased overtime in Brazil though it varied according to the evaluated cities. In concordance, other studies have shown an increase in awareness and willingness to use PrEP in Brazil from 2014 to 2018 [10–12, 16]. This is likely the result of the expansion of PrEP demonstration projects to other cities (e.g. inclusion of Manaus and Porto Alegre to PrEP Brasil study) [59], the initiation of ImPrEP demonstration study in 12 Brazilian cities (http://imprep.org/) and the campaigns to increase PrEP knowledge among key populations in websites (e.g. http://prepbrasil.com.br), social media (e.g. http://facebook.com/prepbrasil) and apps for sexual encounters. That being said, a qualitative study conducted in 2016 in Salvador, Bahia, in the Northeast of Brazil, showed limited knowledge and willingness to use PrEP in their population and highlighted the importance of raising knowledge on the benefits and possible adverse events following PrEP uptake [60]. Acknowledging the heterogeneity in PrEP awareness is important as PrEP is being offered at no cost to high-risk populations in the Brazilian Public Health System. Increased efforts and resources in particular cities or regions are paramount to increase awareness and create demand for PrEP.

This study has limitations. First, web-based studies are not probabilistic sampling strategies, precluding the generalization of the findings to all Brazilian MSM. Considering this is an online convenience sample, geographic comparisons should be analyzed with caution. Moreover, our findings are based on MSM who have access to cellphones and who use GSN apps or social media so it is not generalizable to all MSM in Brazil. Recent data from the Brazilian Institute of Geography and Statistics suggests that 76% of the Brazilian population has access to internet connection [17]. Given the cross-sectional nature of the data, causality and the direction of association may not be inferred. All collected data were self-reported by participants and may be subject to bias. However, individuals tend to be more open and honest through web-based surveys, thereby reducing the possibility of social desirability bias [61]. Our data are subject to recall bias due to 6-month or 12-month recall periods. There is also a concern about participants taking the survey multiple times. To mitigate this bias, the first question of the survey was, “Are you answering this survey for the first time?”. On the definition of our high-risk behavior outcomes, we used three different definitions that have been consistently used in other studies as well as in guidelines to define populations at substantial risk of HIV infection [14–16, 62–64]. The use of the HIRI-MSM as the outcome in the regression model precluded the evaluation of stimulants use as a covariate as it is already included in the scale. Instead, we focused on the effect of alcohol use as measured by binge drinking. Finally, we did not collect data on “sex with HIV-infected partners on antiretroviral treatment with undetectable viral load” and thus could not explore how this might have impacted the association of HIV perceived risk with sexual behavior.

## Conclusions

Overall, MSM socio-demographic characteristics were heterogeneous among Brazilian cities, but similarities were noted among the cities from the same administrative region with a marked exception of the Federal District not following the patterns for the Central-West region. Some behaviors were more homogeneous across the country, including high-risk sexual behavior, though never testing for HIV was notably higher in the least developed cities. Combination HIV prevention is most needed among young men who self-identify as gay/homosexual, report binge drinking or prior STI.

## Supplementary information

**Additional file 1.** Brazilian MSM Survey Instrument. The survey instrument is composed of five sections (25 questions) addressing: sociodemographic information, substance use, sexual behavior and history of sexually transmitted infections, HIV perceived risk and use of HIV testing as well as awareness and willingness to use PrEP.

## Data Availability

The datasets analyzed during the current study are available from the corresponding author on reasonable request.

## Abbreviations

AIDS: acquired immunodeficiency syndrome
CDC: Centers for Disease Control and Prevention
CI: confidence interval
FTC/TDF: emtricitabine and tenofovir disoproxil fumarate
GDP: gross domestic product
GSN: geosocial networking
HDI: human development index
HIRI-MSM: HIV Incidence Risk Index for MSM
HIV: human immunodeficiency virus
IQR: interquartile range
LGBTQI: lesbian, gay, bisexual, transgender, queer and intersex
MSM: gays, bisexuals and other men who have sex with men
MW: minimum wage
OR: odds ratio
PrEP: pre-exposure prophylaxis
STI: sexually transmitted infections
SUS: Brazilian Public Health System
