# Supplementary material for: How heterogeneous are MSM from Brazilian cities? An analysis of sexual behavior and perceived risk and a description of trends in awareness and willingness to use pre-exposure prophylaxis
Source: BMC Infect Dis. 2019 Dec 19;19:1067. doi: 10.1186/s12879-019-4704-x (PMC6923868; doi:10.1186/s12879-019-4704-x)
Supplement: Supplementary file 1 — Additional file 1. Brazilian MSM Survey Instrument. The survey instrument is composed of five sections (25 questions) addressing: sociodemographic information, substance use, sexual behavior and history of sexually transmitted infections, HIV perceived risk and use of HIV testing as well as awareness and willingness to use PrEP. [file 12879_2019_4704_MOESM1_ESM.docx]

brazilian MSM survey INSTRUMENT

1. **To which gender identify do you most identify?**

- Cisgender man
- Cisgender woman 🡪 stop the survey
- Transgender man 🡪 stop the survey
- Transgender woman 🡪 stop the survey
- Travesti 🡪 stop the survey
- Other genders: ________________ 🡪 stop the survey

1. **Is this the first time you complete this questionnaire?**

- Yes
- No 🡪 stop the survey

1. **Where did you hear about this survey?**

- Grindr
- Facebook
- Hornet
- Other. Please, complete: ________________

1. **In which city do you live?**

- Aracajú (SE)
- Belém (PA)
- Belo Horizonte (MG)
- Boa Vista (RR)
- Brasília (DF)
- Campinas (SP)
- Campo Grande (MS)
- Cuiabá (MT)
- Curitiba (PR)
- Duque de Caxias (RJ)
- Fortaleza (CE)
- Florianópolis (SC)
- Goiânia (GO)
- João Pessoa (PB)
- Macapá (AP)
- Maceió (AL)
- Manaus (AM)
- Natal (RN)
- Niterói (RJ)
- Palmas (TO)
- Porto Alegre (RS)
- Porto Velho (RO)
- Recife (PE)
- Rio Branco (AC)
- Rio de Janeiro (RJ)
- Salvador (BA)
- Santos (SP)
- São Luís (MA)
- São Paulo (SP)
- Teresina (PI)
- Vitória (ES)
- Other city: ____________________________________

1. **How old are you?**

years 🡪 If under 18 years, stop the questionnaire

1. **What is your highest level of education?**

- No education
- Preliminary school (1-4 years)
- Primary school (5-9 years)
- Seconday school (10-12 years)
- Undergraduation (13-16 years)
- Graduation (>16 years)

1. **What is your race?**

- Black
- Pardo (Mix black)
- White
- Native
- Asian

1. **What is your montly family income?**

- No income
- 1 minimum wage or less
- 1 to 3 minimun wages
- 3 to 10 minimum wages
- More than 10 minimun wages

1. **What is your sexual orientation?**

- Bisexual
- Homosexual
- Heterosexual
- Other: _________________
- I don’t want to answer

1. **Do you have a steady partner?**

- No
- Yes, male
- Yes, female

1. **Do you use apps or websites to search male partners for sex?**

- Never
- Once a month
- Once a week
- Only during the weekends
- Daily

1. **In the last 6 months, did you use stimulants (cocaine, poppers, crack, or amphetamines)?**

- Yes
- No

1. **In the last 6 months, did you drink 5 or more drinks in a couple of hours?**

- Yes
- No

1. **Have you ever been tested for HIV?**

- No
- Yes, at least once in lifetime

1. **Why have you never tested for HIV?**

- I am not at risk of getting infected
- I don’t think it is practical to go to a health care center
- I am ashamed
- I am afraid of getting a positive result
- I am too lazy
- I don’t want to answer
- Other: _____________________________________

1. **Have you ever tested positive for HIV?**

- Yes🡪 stop the questionnaire
- No
- I don’t want to answer

1. **In your opinion, what is your risk of getting HIV in the next year?**

- No risk
- Low risk
- High risk/50%
- Certain/100%
- I don’t know or I don’t want to answer

1. **In the previous 6 months, how many male partners did you have sex with?**

- No male partner
- 1 to 5
- 6 to 10
- More than 10

1. **In the previous 6 months, how many times did you have condomless receptive anal sex?**

- None
- One or more

1. **In the previous 6 months, how many times did you have condomless insertive anal sex?**

- None
- One or more

1. **In the previous 6 months, how many HIV-positive male partners did you have sex with?**

- No partner
- Only one partner
- More than one partner

1. **In the previous 6 months, how many times did you have insertive anal intercourse with HIV-positive partners?**

- None
- 1 to 4 times
- 5 or more times

1. **In the last 6 months, were you diagnosed with sexually transmitted infections (STI; syphilis, gonorrhea or rectal chlamydia)?**

- Yes
- No

*Preexposure profilaxis (PrEP) is the use of antiretrovirals once daily to prevent HIV infection. PrEP is now recommended by the World Health Organization (WHO) and the Brazilian Ministry of Health. Taking PrEP requires going to the health care center at each 3 months for HIV and STI tests.*

1. **Have you ever heard of PrEP?**
   - Yes
   - No
2. **What level of interest would you have in using PrEP?**
   - High interest
   - Some interest
   - Low interest
   - No interest
